# Supplementary material for: Ontology-guided segmentation and object identification for developmental mouse lung immunofluorescent images
Source: BMC Bioinformatics. 2021 Feb 23;22:82. doi: 10.1186/s12859-021-04008-8 (PMC7901098; doi:10.1186/s12859-021-04008-8)

Supplementary Figure 1: Comparison of results between a) our odifmap method and b) Mask R-CNN.


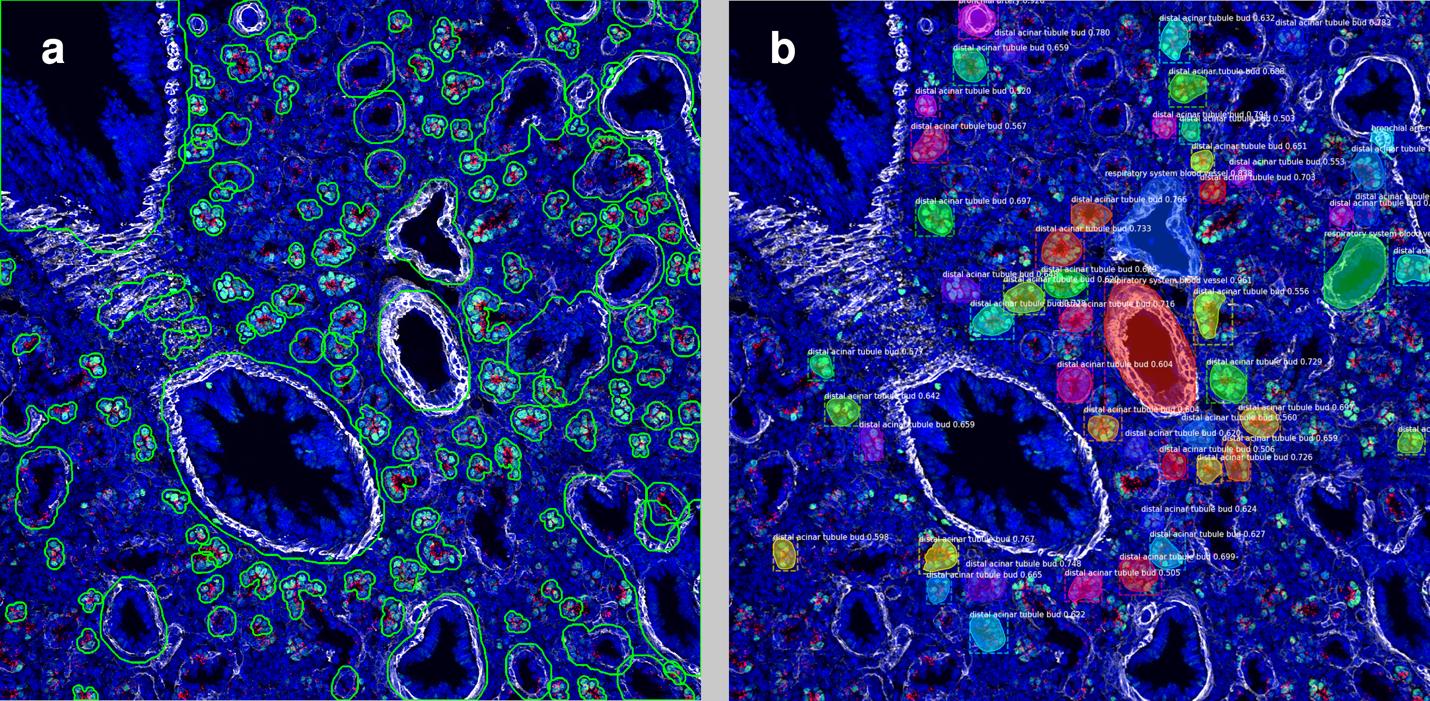


Supplementary Figure 2. Screenshot from Protégé showing the ontology relations for the distal acinar tubule bud.

*
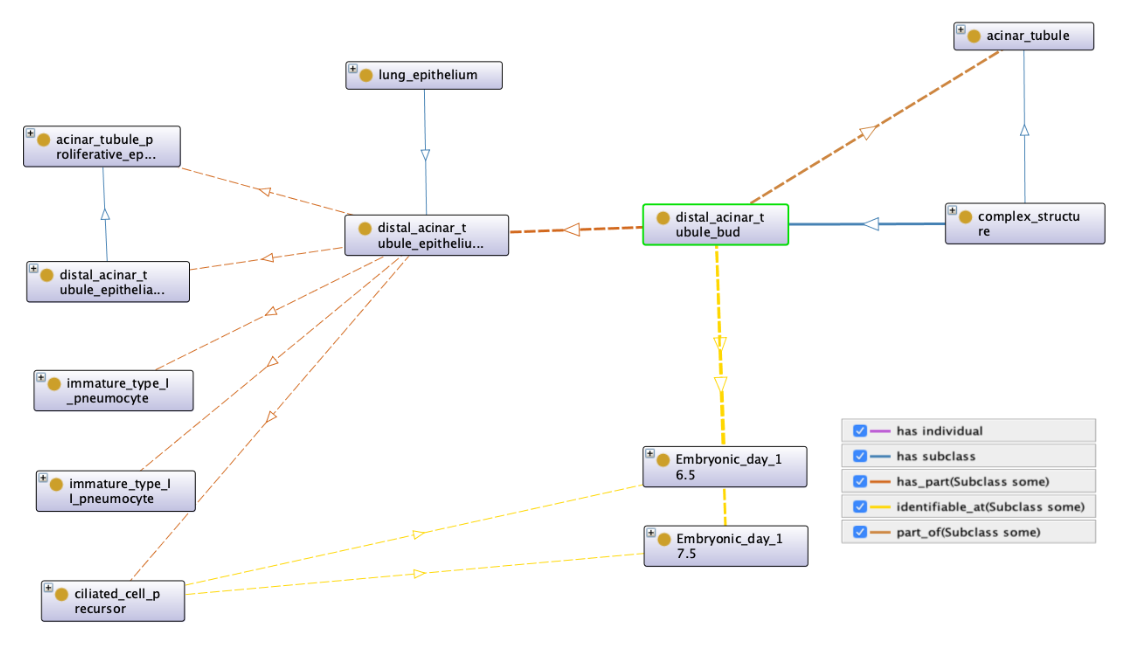
*

Supplementary Figure 3. Example segmentation configuration annotated with descriptions of available options.


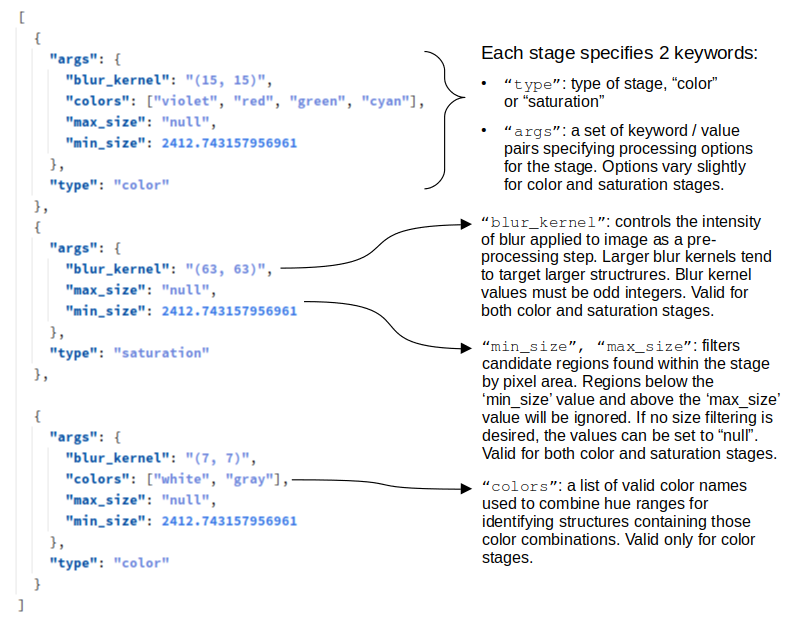


Supplementary Table 1: Confusion matrices comparing our odifmap method (top) with Mask R-CNN (bottom).


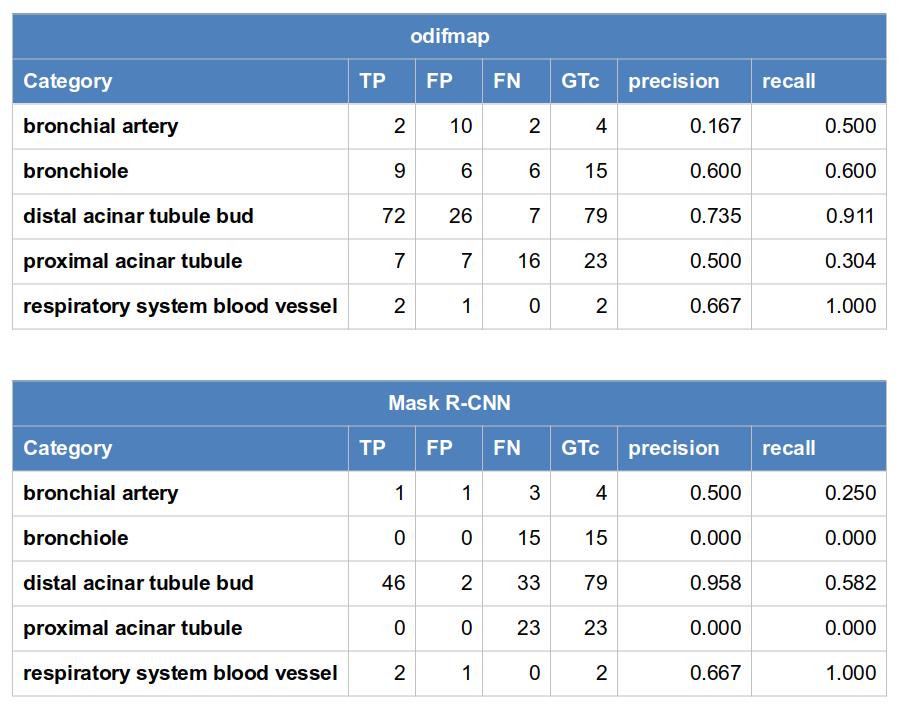


Supplementary Table 2: a) Represents a list of domain ontologies and the number of terms from each of those ontologies used in the lung application ontology. b) represents a list of relations and the relative ontology name and ID that have been used in the lung application ontology.


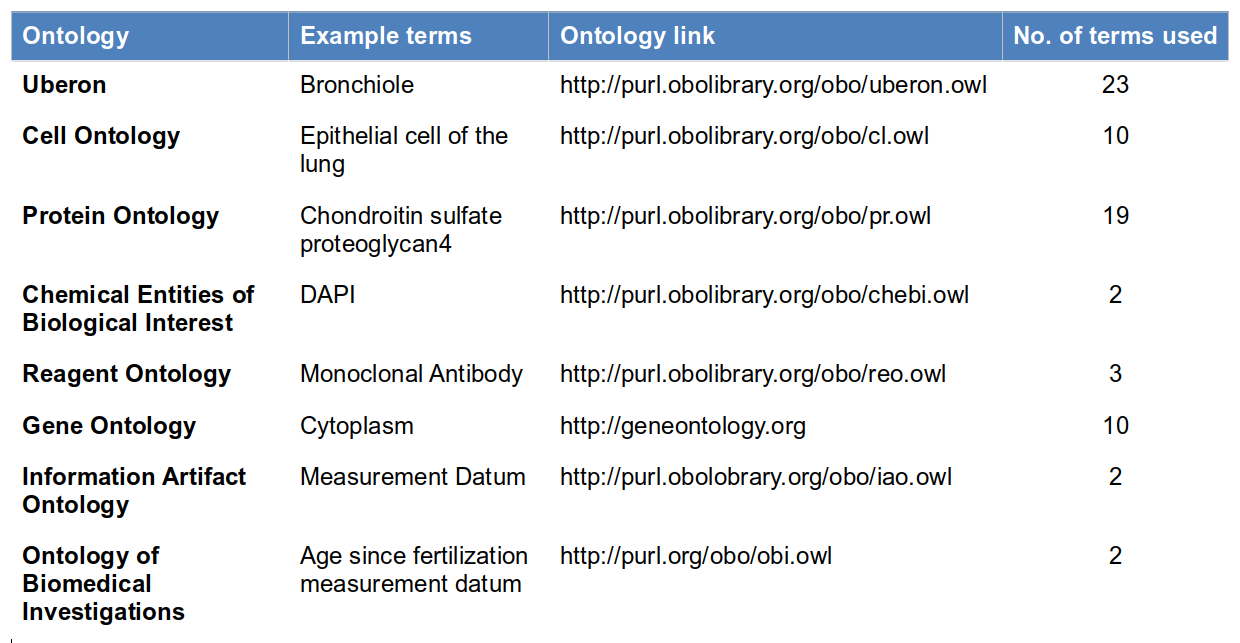


b)


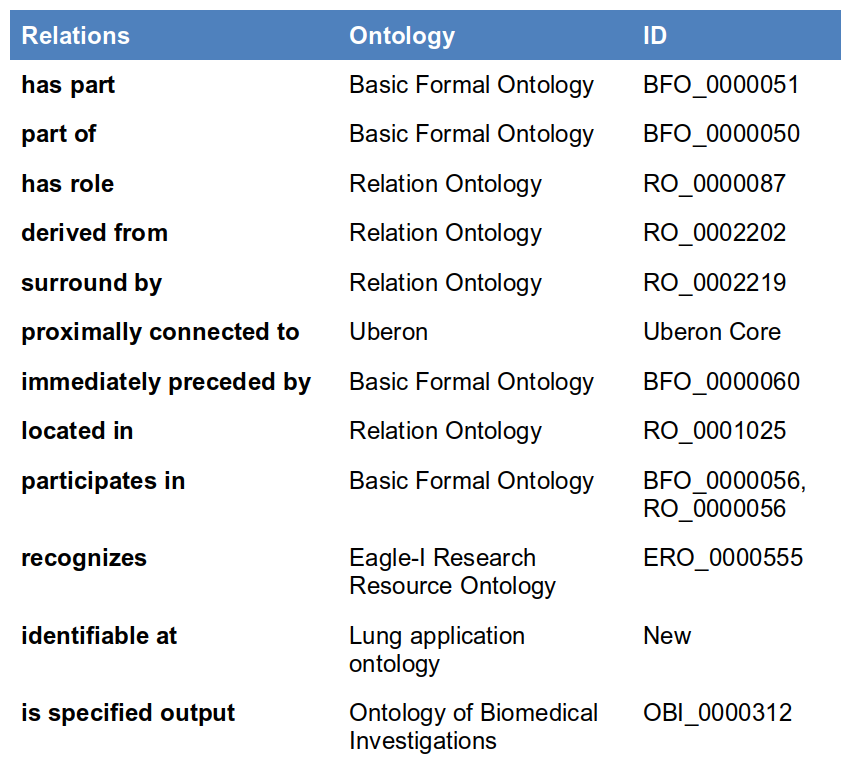


Supplemental Table 3: Ontology query results for the probes present in an image, demonstrating the linkage between probes and anatomical structures, as well as their contextual information regarding location where they are present in the structure.


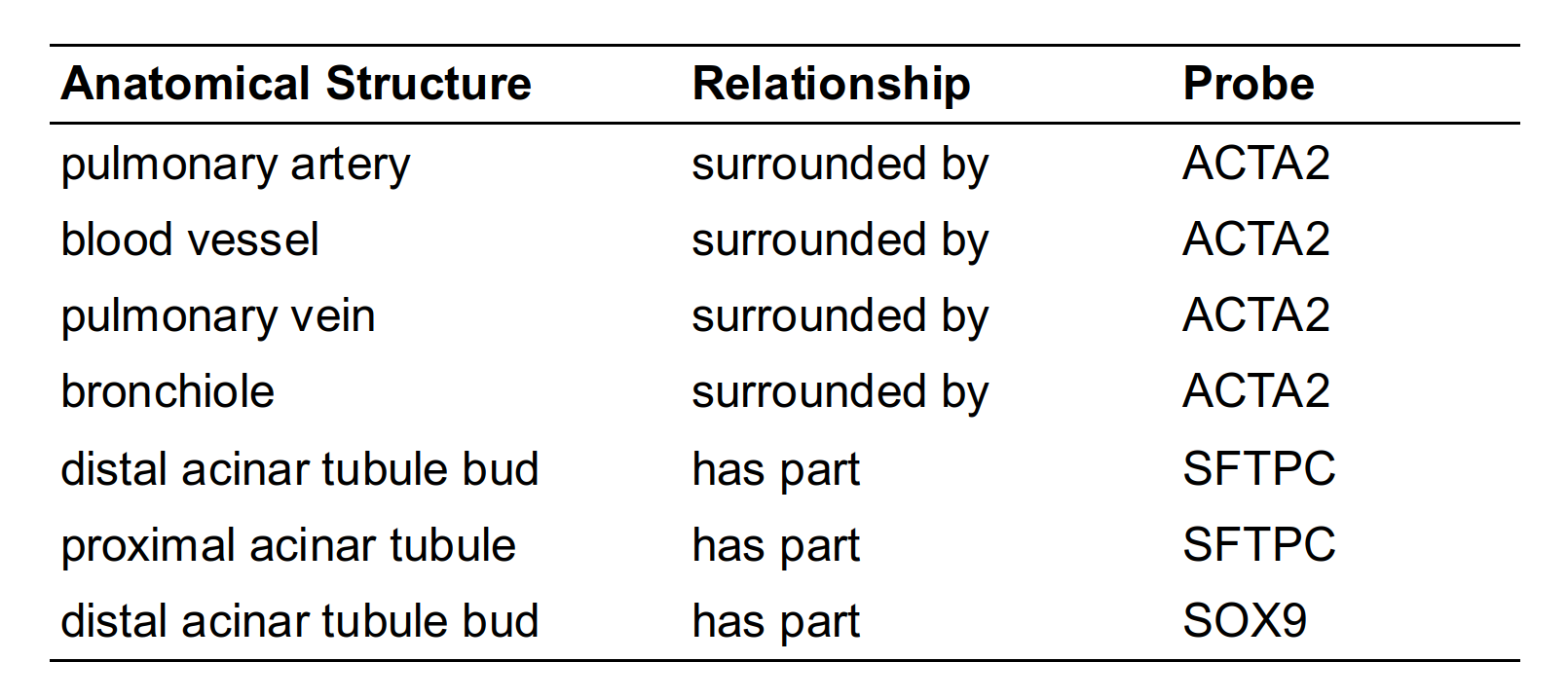


Supplementary Table 4: Color definitions utilized in the image analysis pipeline. Colors are defined by partitioning the complete HSV color space such that each HSV value occurs in only a single color label. The hue range spans 180 values, saturation spans 256 values, and value (intensity) spans 256 values. Color labels are classified into 3 groups: major colors spanning 40 hue values, minor colors spanning 20 hue values, and monochrome colors.


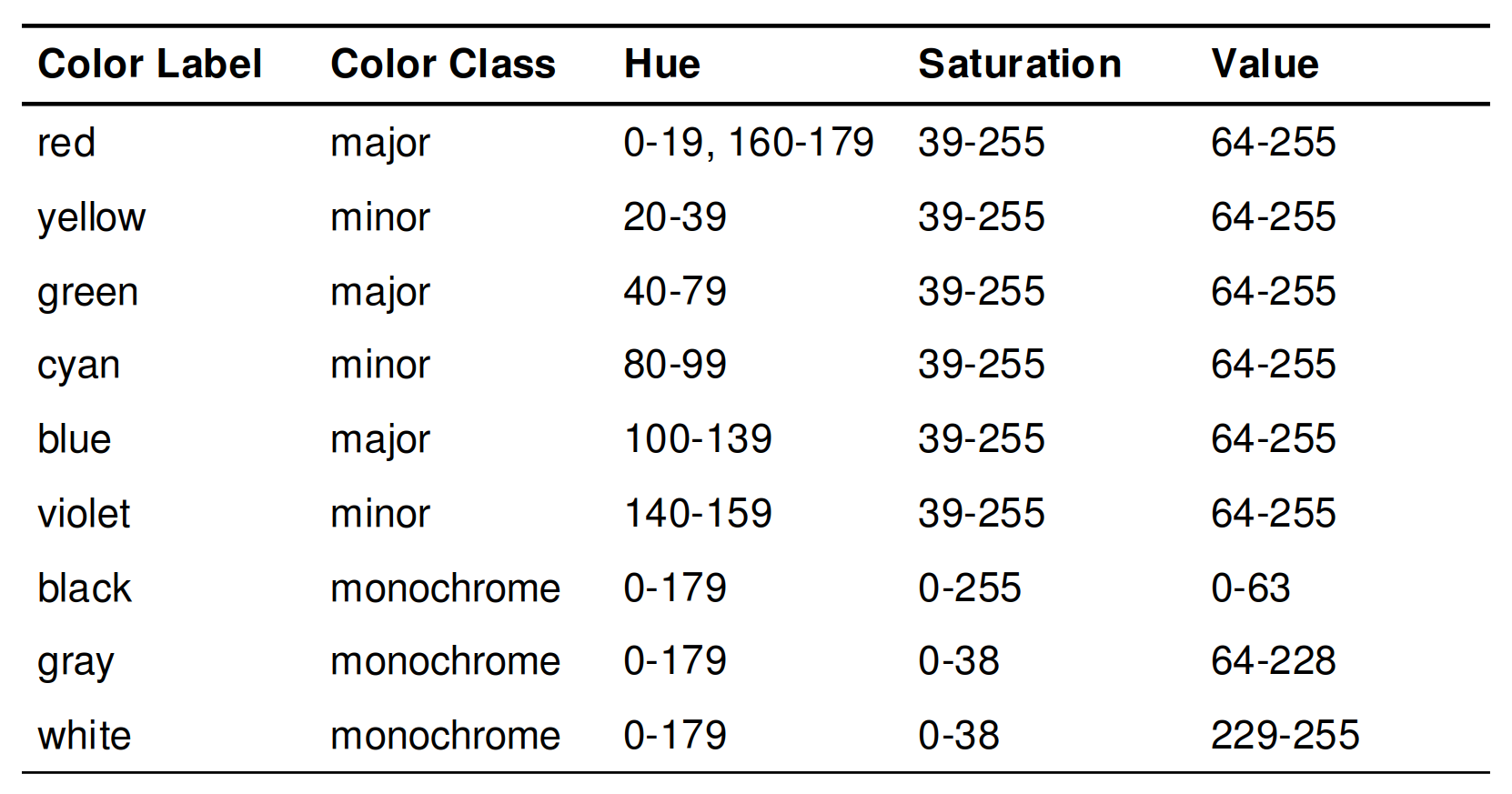

Supplement: Supplementary file 1 — Additional file 1: Supplementary Figure 1. Comparison of results between a) our odifmap method and b) Mask R-CNN. Supplementary Figure 2. Screenshot from Protégé showing the ontology relations for the distal acinar tubule bud. Supplementary Figure 3. Example segmentation configuration annotated with descriptions of available options. Supplementary Table 1. Confusion matrices comparing our odifmap method (top) with Mask R-CNN (bottom). Supplementary Table 2. a) Represents a list of domain ontologies and the number of terms from each of those ontologies used in the lung application ontology. b) represents a list of relations and the relative ontology name and ID that have been used in the lung application ontology. Supplemental Table 3. Ontology query results for the probes present in an image, demonstrating the linkage between probes and anatomical structures, as well as their contextual information regarding location where they are present in the structure. Supplementary Table 4. Color definitions utilized in the image analysis pipeline. Colors are defined by partitioning the complete HSV color space such that each HSV value occurs in only a single color label. The hue range spans 180 values, saturation spans 256 values, and value (intensity) spans 256 values. Color labels are classified into 3 groups: major colors spanning 40 hue values, minor colors spanning 20 hue values, and monochrome colors. [file 12859_2021_4008_MOESM1_ESM.docx]
